# Supplementary material for: Longitudinal Effects of Lipid-Lowering Treatment on High-Risk Plaque Features and Pericoronary Adipose Tissue Attenuation Using Serial Coronary Computed Tomography
Source: Diagnostics (Basel). 2025 Sep 16;15(18):2340. doi: 10.3390/diagnostics15182340 (PMC12468858; doi:10.3390/diagnostics15182340)

Suppl. Table 1: Number of baseline and follow-up CCTA examinations performed with each scanner type. In addition. scanner specific parameters are provided.

Scanner vendor/type / CCTA parameter		Siemens			Philips			GE Healthcare
		Somatom Definition AS+	Somatom Definition Flash	Somatom Force	Spectral CT 7500	IQon Spectral CT	Brilliance CT 6000 (iCT)	Revolution CT
Number of examinations	Baseline	12	33	104	0	5	41	21
	Follow-up	12	19	118	4	19	23	21
Slices	Number	128	2x128	384	2x256	2x128	256	256
	Thickness	0.69	0.73	0.58	0.69	0.72	0.84	0.63
Tube	Current [mA range]	20-800	250-500	290-560	10-1000	10-1000	10-1000	1200
	Voltage [kV range]	70-140	70-140	70-150	80-140	80-140	80-140	70-140
Resolution	Temporal [ms]	150	75	66	135	135	135	140
	Spatial [mm]	0.33	0.3	0.24	16lp/cm	16lp/cm	24lp/cm	230
Table feed [mm/s]		192	430	737	600	185	185	300
Max pitch		1.5	1.8	3.2	1.65	1.8	1.8	1.531:1
Minimal gantry rotation time [ms]		300	280	250	270	270	270	280
Iterative reconstruction		SAFIRE	SAFIRE	ADMIRE	iDose^4 / IMR	iDose^4 / IMR	iDose^4 / IMR	ASiR-V / TrueFidelity

Suppl. Table 2: Associations between changes in PCAT and plaque volumes or DPFS

Changes in plaque volumes versus absolute changes in PCAT:

**Correlation coefficient r**	**-0.036**
**Significance level**	P=0.66
**95% Confidence interval for r**	-0.19 to 0.12

Changes in plaque volumes versus relative changes in PCAT:

**Correlation coefficient r**	**0.038**
**Significance level**	P=0.64
**95% Confidence interval for r**	-0.12 to 0.20

DPFS versus absolute changes in PCAT:

**Correlation coefficient r**	**-0.021**
**Significance level**	P=0.79
**95% Confidence interval for r**	-0.18 to 0.14

DPFS versus relative changes in PCAT:

**Correlation coefficient r**	**0.025**
**Significance level**	P=0.76
**95% Confidence interval for r**	-0.13 to 0.18

Suppl. Table 3 A-B. Univariable models for the prediction of high-risk plaque feature regression (A) and progression (B) based on treatment with aspirin or P2Y12 inhibitors and the number of antihypertensive medications per patient.

A. Regression of high-risk plaque features	Coefficient	Standard error	Wald	Hazard ratio	95% CI	p-values
Aspirin or P2Y12 inhibitors	0.11	0.29	0.14	1.31	0.75 to 2.28	0.70
Number of antihypertensive medications per patient	0.11	0.12	0.85	1.12	0.87 to 1.43	0.35
B. Progression of high-risk plaque features						
Aspirin or P2Y12 inhibitors	0.27	0.28	0.91	1.31	0.75 to 2.28	0.33
Number of antihypertensive medications per patient	0.26	0.12	4.46	1.30	1.01 to 1.66	0.03

Suppl. Table 4 A-B. Cox-proportional hazard regression models for the prediction of high-risk plaque feature regression (A) and progression (B), including the number of antihypertensive medications as an additional variable.

A. Regression of high-risk plaque features	Coefficient	Standard error	Wald	Hazard ratio	95% CI	p-values
Age	0.00	0.021	0.049	1.00	0.96 to 1.04	0.82
Total number of CV risk factors	-0.33	0.22	2.11	0.71	0.45 to 1.12	0.14
Baseline plaque volume (mm^3)	0.001	0.0003	17.25	1.001	1.000 to 1.002	<0.0001
Lipid-lowering treatment intensity	0.91	0.27	11.01	2.48	1.45 to 4.25	<0.001
Baseline $PCAT_{RCA}$	-0.014	0.018	0.57	0.98	0.95 to 1.02	0.44
Number of antihypertensive medications per patient	0.13	0.19	0.50	1.14	0.78 to 1.67	0.47
**B. Progression of high-risk plaque features**						
Age	0.008	0.026	0.11	1.00	0.95 to 1.06	0.73
Total number of CV risk factors	-0.15	0.22	0.46	0.85	0.55 to 1.33	0.49
Baseline plaque volume (mm^3)	-0.0001	0.0007	0.03	0.99	0.99 to 1.00	0.85
Lipid-lowering treatment intensity	0.13	0.36	0.14	1.14	0.56 to 2.34	0.70
Baseline $PCAT_{RCA}$	0.031	0.013	5.50	1.03	1.00 to 1.05	0.02
Number of antihypertensive medications per patient	0.38	0.22	2.80	1.46	0.93 to 2.29	0.09

Suppl. Table 5. Different key clinical markers in patients with and without PCAT

Age	n	Mean	SD
(1) 0	69	63,3623	10,0469
(2) 1	147	62,9782	9,637

F-ratio	0,0726
Significance level	P = 0,788

CV risk factors	n	Mean	SD
(1) 0	69	1,8986	1,0452
(2) 1	147	2,0068	1,1134

F-ratio	0,461
Significance level	P = 0,498

Baseline CCTA plaque volumes	n	Mean	SD
(1) 0	69	2,3768	1,1389
(2) 1	147	2,5578	1,1111

F-ratio	1,226
Significance level	P = 0,269

Suppl. Figure S1: Study patient flowchart

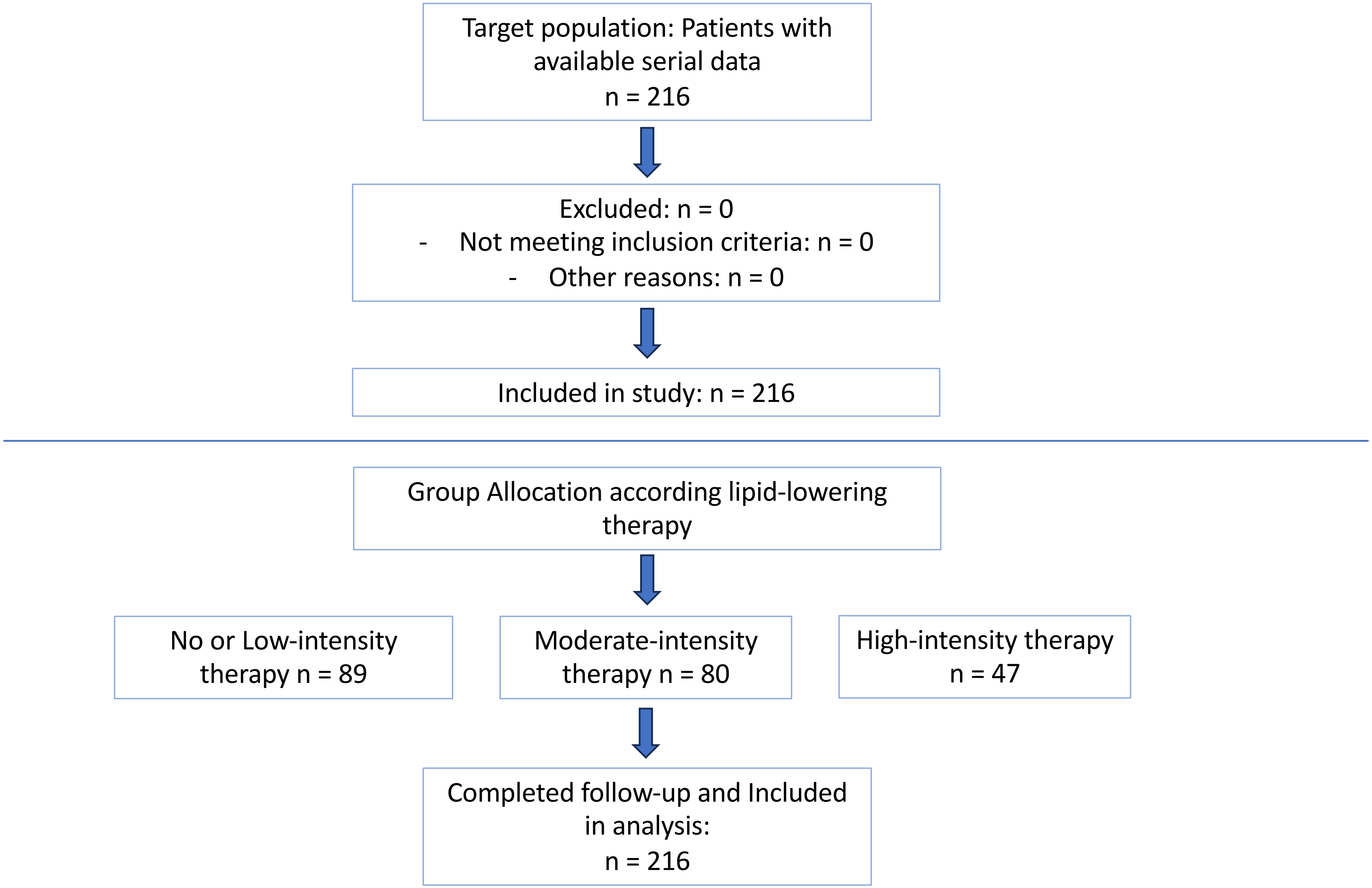

Suppl. Figure S2: High-risk plaque feature regression probability on different intensity of lipid-lowering treatment

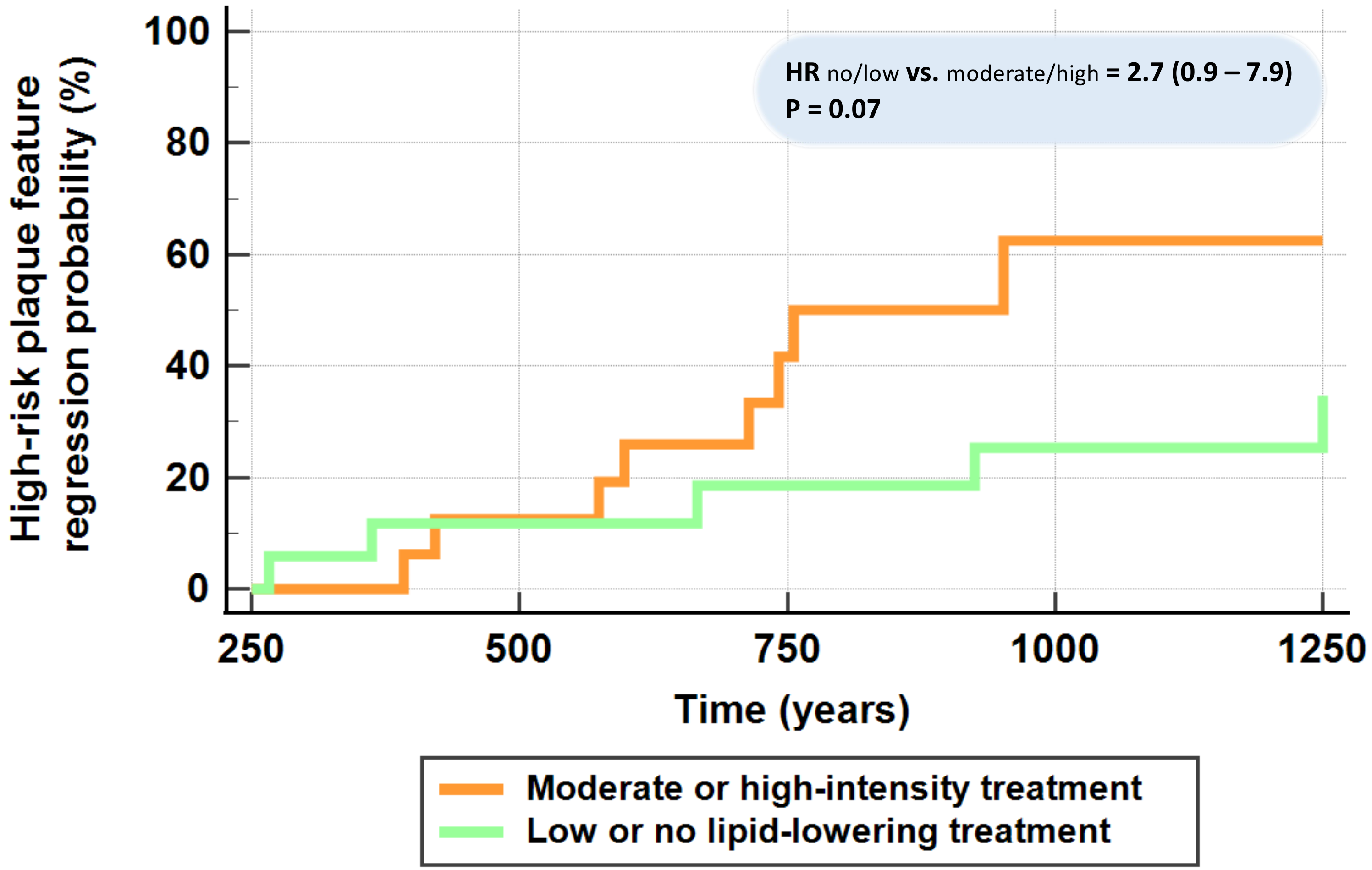

Suppl. Figure S3: ROC-analysis on relative changes in non-calcified plaque volumes within randomly selected time intervals

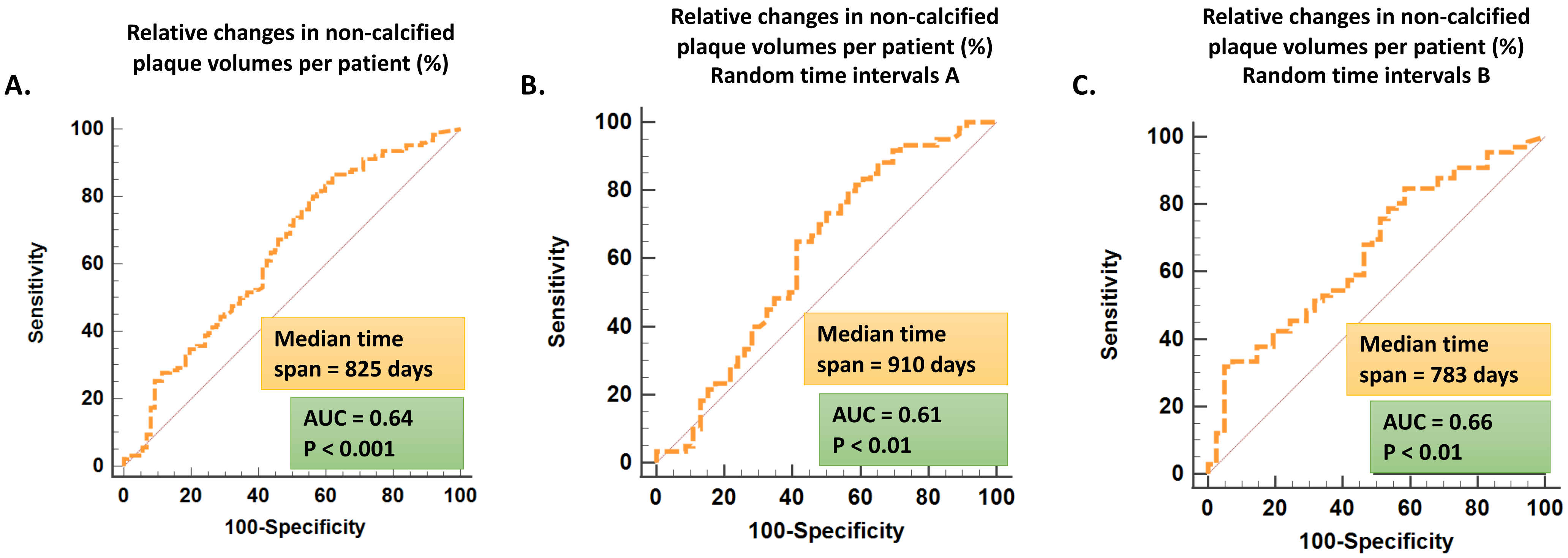

Suppl. Figure S4: Complex interactions between plaque progression and regression over time. Role of PCAT and lipid-lowering medications.

Plaque ´delipidisation´

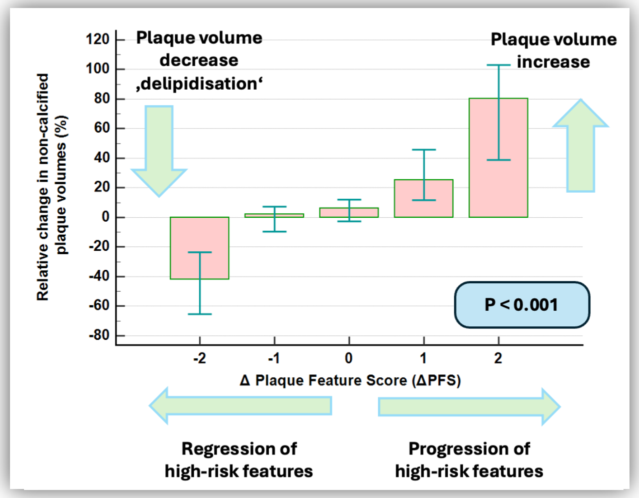

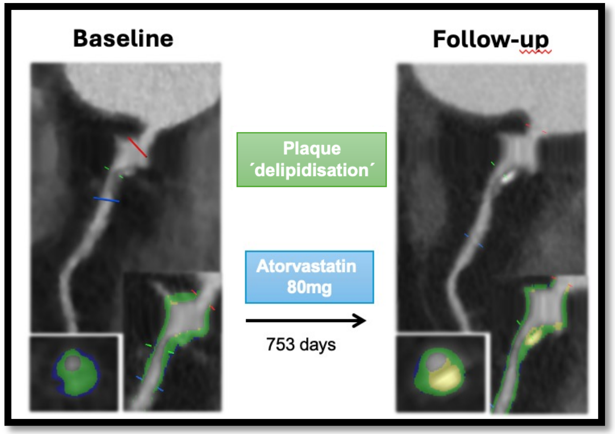

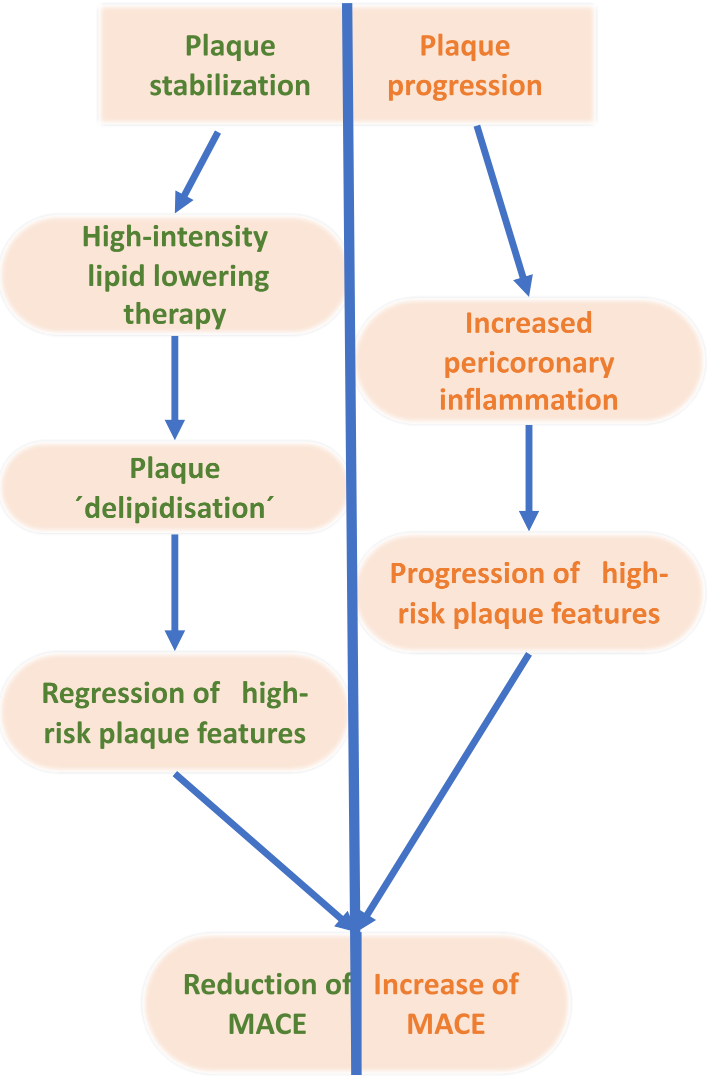

Progression of high-risk features triggered by increased inflammation

Progression of high-risk plaque features	Odds ratio	p-values
Age	1.00	0.82
Total number of CV risk factors	0.87	0.57
Baseline PCAT $_{RCA}$	1.02	0.03

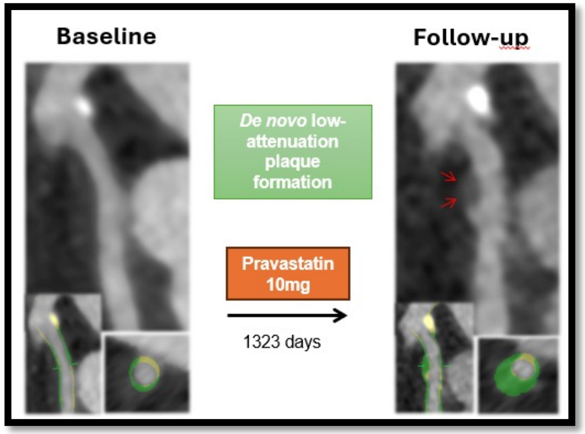

Suppl. Figure S5: Time range between the 1st and 2nd CCTA scan.

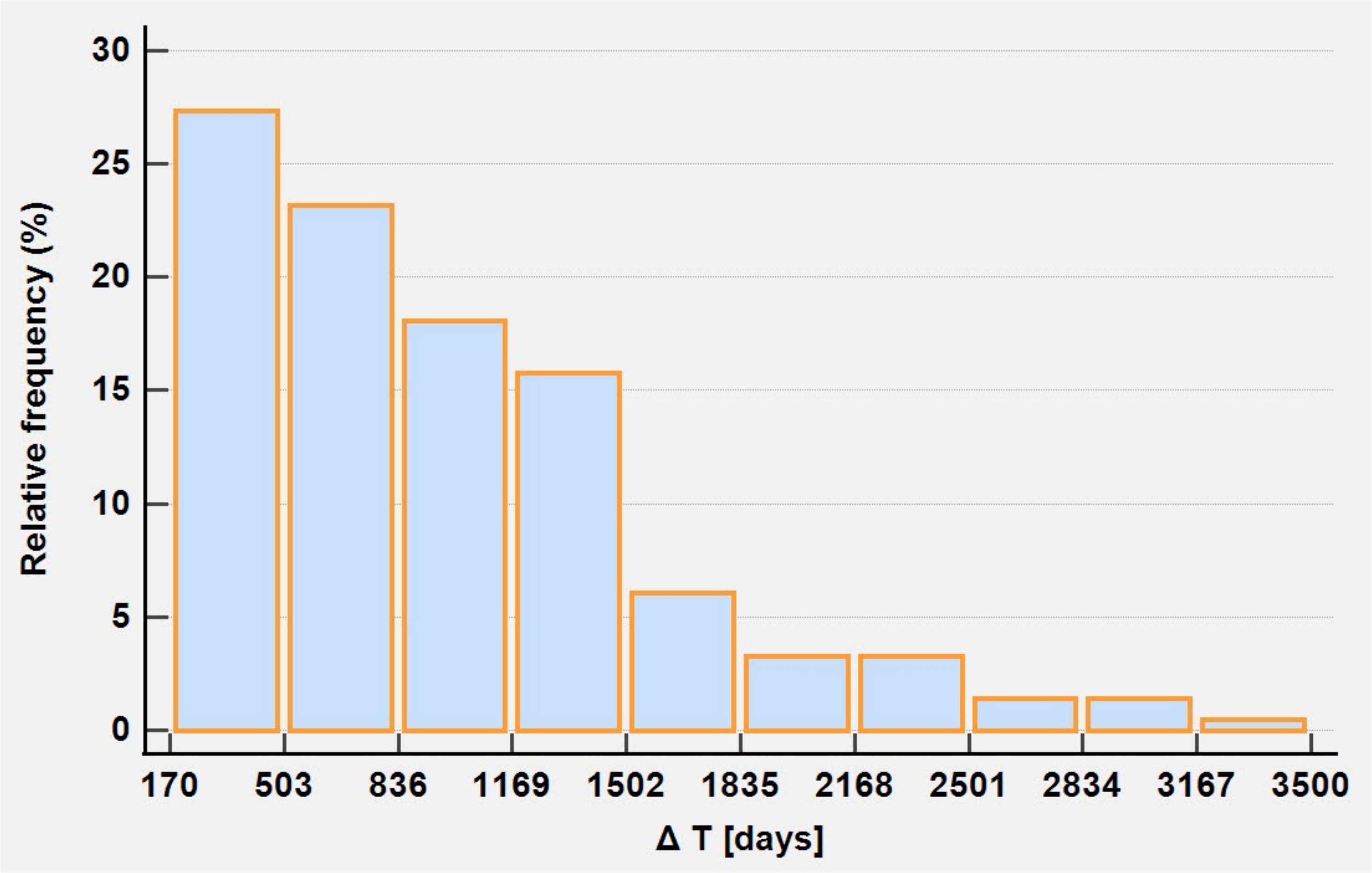

Supplement: Supplementary file 1 [file diagnostics-15-02340-s001.zip › diagnostics-3819051-supplementary.pdf]
